# Nature of Protein Family Signatures: Insights from Singular Value Analysis of Position-Specific Scoring Matrices

**DOI:** 10.1371/journal.pone.0001963

**Published:** 2008-04-09

**Authors:** Akira R. Kinjo, Haruki Nakamura

**Affiliations:** Institute for Protein Research, Osaka University, Suita, Osaka, Japan; University College London, United Kingdom

## Abstract

Position-specific scoring matrices (PSSMs) are useful for detecting weak homology in protein sequence analysis, and they are thought to contain some essential signatures of the protein families. In order to elucidate what kind of ingredients constitute such family-specific signatures, we apply singular value decomposition to a set of PSSMs and examine the properties of dominant right and left singular vectors. The first right singular vectors were correlated with various amino acid indices including relative mutability, amino acid composition in protein interior, hydropathy, or turn propensity, depending on proteins. A significant correlation between the first left singular vector and a measure of site conservation was observed. It is shown that the contribution of the first singular component to the PSSMs act to disfavor potentially but falsely functionally important residues at conserved sites. The second right singular vectors were highly correlated with hydrophobicity scales, and the corresponding left singular vectors with contact numbers of protein structures. It is suggested that sequence alignment with a PSSM is essentially equivalent to threading supplemented with functional information. In addition, singular vectors may be useful for analyzing and annotating the characteristics of conserved sites in protein families.

## Introduction

Protein sequence alignment using a position-specific scoring matrix (PSSM) or sequence profile [1], [2] is now a standard tool for sequence analysis[3], [4]. Using a PSSM, it is often possible to detect very distantly related proteins which cannot be detected by the standard pairwise alignment based on a position-independent amino acid substitution matrix (AASM).

An AASM is a 20×20 real (usually symmetric) matrix each element of which reflects the tendency of substitution between amino acid residues. There have been many kinds of AASMs developed to date among which the most popular ones include the PAM [5] and the BLOSUM series [6]. General properties of AASMs are now well clarified[7], [8], [9], [10]. Tomii and Kanehisa found that the PAM matrices can be well approximated by the volume and hydrophobicity of amino acid residues[8]. A similar result was obtained by Pokarowski et al.[10], but they also pointed out the importance of the coil preferences of amino acids residues. Using eigenvalue decomposition, Kinjo and Nishikawa[9] showed that the most dominant component of AASMs is the relative mutability[5] for closely related homologs, but it changes to hydrophobicity below the sequence identity of 30%, and this transition of dominant modes was related to the so-called twilight zone of sequence comparison[11], [12]. There are also AASMs specifically optimized to overcome the twilight zone [13], [14].

Detection of very distant homologs is often possible by using PSSM-based sequence alignment methods such as PSI-BLAST[4] or hidden Markov models[3], [15] because a PSSM is specific to a particular protein family so that some family-specific features can be exploited. In a PSSM, family-specific features are expressed as position-dependent substitution scores, and hence a PSSM is an *N*×20 matrix where *N* is the length of the protein or protein family it represents. Since PSSMs can be regarded as an extension of sequence motifs[15], family-specific features are, to the first approximation, a pattern of amino acid residues around functionally or structurally important sites expressed in a probabilistic manner. In order to further understand the mechanism by which the effectiveness of PSSMs is realized, however, it is necessary to elucidate more general characteristics of PSSMs that are shared across different protein families.

To delineate the general properties of PSSMs, we analyze them by using singular value decomposition (SVD). By applying SVD, a PSSM can be decomposed into 20 orthogonal components of varying importance. Each singular component consists of a singular value (a scalar), right singular vector (r-SV) and left singular vector (l-SV). A singular value represents the relative importance of the component whereas the corresponding r-SV (a 20-vector) represents a property of 20 amino acid types and the l-SV may be regarded as a one-dimensional (1D) numerical representation of the amino acid sequence that is “dual” to the property represented by the r-SV. Since r-SVs can be regarded as amino acid indices[16], [17], [8], we can infer their meaning by comparing them with the entries of the AAindex database[18] which compiles many amino acid indices published to date. This is a natural generalization of a previous work where AASMs were analyzed by using eigenvalue decomposition [9]. The present analysis revealed a tendency of PSSMs that is analogous to the AASMs for close homologs. That is, the first principal component disfavors any substitutions and potentially functionally important residues are more severely penalized, and the second component is highly correlated with sequence and structural properties related to hydrophobicity. These features are expected to contribute to the effectiveness of sequence alignment based on PSSMs.

## Methods

### Singular value decomposition of position-specific scoring matrix

**Figure 1:**
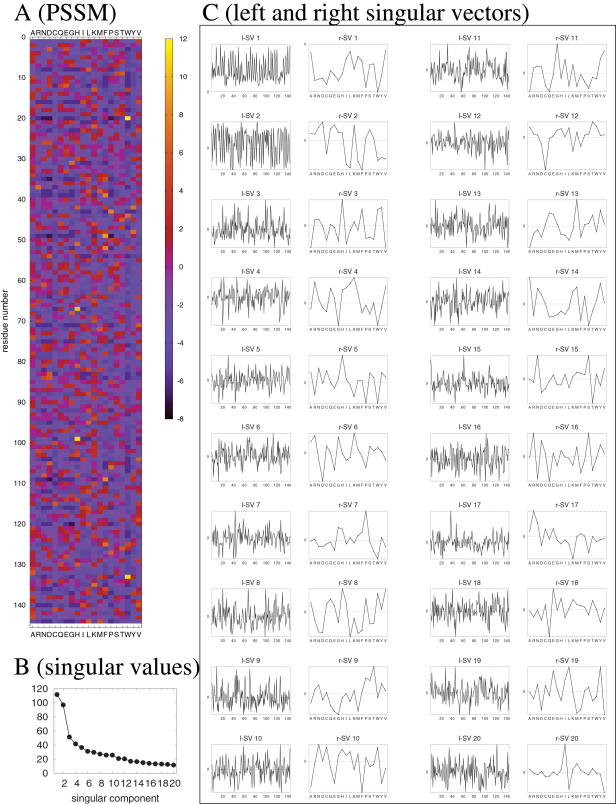
Example of singular value decomposition[19] of a PSSM (c.f. Eq. 1). A: The original PSSM (based on the PDB entry 3sdhA [44]); B: Singular values; C: Pairs of left singular vector (l-SV) u_α_ of *N* dimensions and right singular vector (r-SV) v_α_ of 20 dimensions (α = 1,…,20). The abscissa indicates residue number for the left singular vectors (l-SV), and amino acid type for the right singular vectors (r-SV). The ordinate shows the vector elements relative to zero (note that only the relative values, not absolute ones, are meaningful).

A position-specific scoring matrix (PSSM) is a real rectangular matrix of size *N*×20 where *N* is the length of the amino acid sequence of a protein (or protein family). We assume *N*>20 although this condition is not strictly necessary. Each column of a PSSM corresponds to an amino acid type, whereas each row corresponds to a site in the amino acid sequence. Let *M* = (*M_ij_*) be a PSSM. The element *M_ij_* represents the score for the amino acid *j* at the site *i* (Fig. 1A). By applying singular value decomposition [19] (SVD), we have
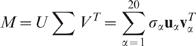
(1)where *U* = (**u**_1_,…,**u**_20_) and *V* = (**v**_1_,…,**v**_20_) are *N*×20 and 20×20 orthogonal matrices, respectively, that is, 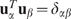 and 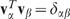 (δ_αβ_ is Kronecker's delta). An example of SVD of a PSSM is given in Fig. 1. The 20-vectors **v**_α_'s are called right singular vectors (r-SV, Fig. 1C). Since each element of a right singular vector numerically represents some property of an amino acid type, we can regard a right singular vector of a PSSM as an amino acid index [16], [17], [8] (possibly specific to the parent PSSM). The *N*-vectors **u**_α_'s are called left singular vectors (l-SV, Fig. 1C). Since each element of a left singular vector numerically represents some property of the corresponding site in the sequence, we can regard a left singular vector of a PSSM as a generalized 1D structure. Σ = *diag*(σ_1_,…,σ_20_) is a diagonal matrix whose elements are the singular values of the PSSM, sorted in the decreasing order (Fig. 1B). Singular values are always non-negative and their magnitudes represent relative importance of the corresponding singular components (i.e., the pair of right and left singular vectors).

### Data sets

We analyze two sets of PSSMs. One is a representative set derived from the Protein Data Bank (PDB)[20] and the other is the Pfam database[21].

The representative protein chains in the PDB were obtained from the PISCES server [22] with cutoffs of 25% sequence identity, 20% R-factor, 2.0Å resolution and sequence length ranging from 40 to 500. Only the structures determined by X-ray crystallography were used. Those proteins which were classified as all-α, all-β, α/β, α+β, multi-domain, or small proteins according to the SCOP (version 1.71) [23] database were retained. As a result, we obtained 1096 protein chains. For each of these proteins, a PSSM was created by running PSI-BLAST against the UniRef100 protein sequence database (release 12.1) [24] with e-value cutoff of 0.0005 and 3 iterations.

Although Pfam is a database of hidden Markov models of protein families[15], we can regard its entries as PSSMs by using only the scores for matching states. We extracted from Pfam release 22.0 (July 2007) those proteins whose sequence lengths were at least 40 residues, resulting in 8869 protein families.

### Searching AAindex

As mentioned above, each right singular vector (r-SV) can be regarded as an amino acid index, a set of numerical values reflecting some property of amino acid residues. In order to clarify the meaning of each r-SV, we scanned the AAindex database [8], [18] (Release 9.1, August, 2006) which compiles many amino acid indices published to date. For a given α ( = 1, 2, …, 20), the amino acid index that showed the highest correlation to the α-th r-SV of each PSSM were identified. If the absolute value of the correlation coefficient between the index and the r-SV is greater than or equal to 0.6, then the index is counted as significant. Identified indices are sorted according to the number of times they are counted as significant. In Table 1, we summarize the descriptions of the AAindex entries that will be mentioned in the Results section.

**Table 1:** AAindex entries mentioned in the text.

ID	Description	Reference
AURR980119	Normalized positional residue frequency at helix termini C″’	Aurora and Rose (1998)[41]
BASU050101	Interactivity scale obtained from the contact matrix	Bastolla et al. (2005)[32]
BASU050103	Interactivity scale obtained by maximizing the mean of correlation coefficient over pairs of sequences sharing the TIM barrel fold	Bastolla et al. (2005)[32]
BEGF750103	Conformational parameter of beta-turn	Beghin and Dirkx (1975)[27]
BUNA790101	alpha-NH chemical shifts	Bundi and Wuthrich (1979)[43]
CHAM830106	The number of bonds in the longest chain	Charton and Charton (1983)[62]
FAUJ880106	STERIMOL maximum width of the side chain	Fauchere et al. (1988)[63]
FUKS010106	Interior composition of amino acids in intracellular proteins of mesophiles	Fukuchi and Nishikawa (2001)[26]
GRAR740102	Polarity	Grantham (1974)[33]
JOND920102	Relative mutability	Jones et al. (1992)[25]
KLEP840101	Net charge	Klein et al. (1984)[42]
KOEP990101	Alpha-helix propensity derived from designed sequences	Koehl and Levitt (1999)[38]
KYTJ820101	Hydropathy index	Kyte and Doolittle (1982)[28]
LEVM760102	Distance between C-alpha and centroid of side chain	Levitt (1976)[64]
LEVM760105	Radius of gyration of side chain	Levitt (1976)[64]
MIYS990101	Relative partition energies derived by the Bethe approximation	Miyazawa and Jernigan (1999)[31]
OOBM770105	Short and medium range non-bonded energy per residue	Oobatake and Ooi (1977)[65]
SNEP660101	Principal component I	Sneath (1966)[29]
SNEP660103	Principal component III	Sneath (1966)[29]
SWER830101	Optimal matching hydrophobicity	Sweet and Eisenberg (1983)[34]

## Results

### Overview

In order to check to what extent a subset of singular components can explain the original PSSM, we calculated the accumulative contribution ratio of each PSSM. The accumulative contribution ratio up to *k*-th singular value is defined as
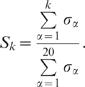
(2)


**Figure 2:**
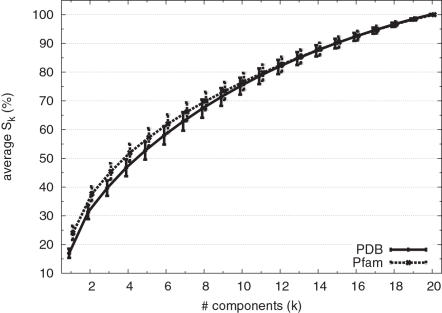
Accumulative contribution ratio (*S_k_*%, *k* = 1,…,20) averaged over the PDB and Pfam sets.

The averages of *S_k_* for *k* = 1,…,20 are shown in Fig. 2. We observe that the first singular value contributes 17% of the total singular values in the PDB set, and 24% in the Pfam set. Thus, the contribution of the first singular component is relatively larger in the Pfam PSSMs than in the PSI-BLAST-generated PSSMs of PDB entries. This tendency may be related to the higher specificity of the Pfam hidden Markov models. 50% contributions are made by first 4 or 5 components in the PDB or Pfam sets, respectively, whereas 90% contributions are made by the first 15 components in the both sets. Compared to the case with AASMs where 50% and 90% contributions are made by first 3 and 10 singular values (or eigenvalues) [9], the “compressibility” of PSSMs is lower in the sense that more components are needed to explain the same fraction (50% or 90%) of the total components. This is a reasonable result since each PSSM should contain some detailed information specific to the family to which the protein sequence belongs, whereas AASMs should contain more general information regarding the patterns of amino acid substitutions shared by many protein families.

**Figure 3:**
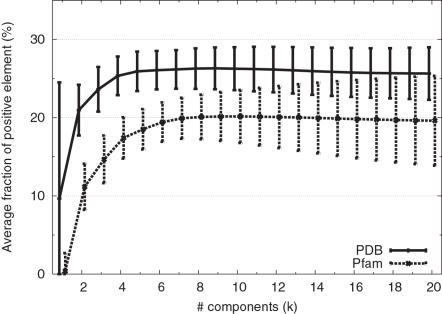
Fraction of positive elements in partial matrices *M_k_* averaged over the PDB and Pfam sets.

In order to glance at the overall characteristics of decomposed PSSMs, we constructed a partial matrix *M_k_* for each PSSM by summing the first *k* components, that is,
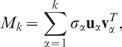
(3)and calculated the fraction of positive elements (*M*_20_ is identical to the original PSSM). In both the PDB and Pfam sets, there are usually more negative elements than positive ones (Fig. 3). This is an expected behavior for log-odds matrices [7]. However, this skewed distribution is greatly pronounced for the *M*_1_ matrices. In fact, most substitutions are disfavored by the first singular component of a PSSM. A typical example is shown in Fig. 1 C where the contribution of the first component (i.e., 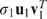) is purely negative. Compared to *M*_1_, other partial matrices (*M_k_* with *k*>1) have more positive elements. This indicates that positive values in the final PSSM must originate from components other than the first one.

### Characteristics of first singular components

In order to interpret the physicochemical or biochemical meaning of the first r-SVs (**v**_1_ in Eq. 1), we scanned the AAindex database and identified amino acid indices that frequently show significant correlations (Table 2).

**Table 2:** Amino acid indices most correlated to the first right singular vectors [frequency (%) in the parentheses].

rank	PDB	Pfam
1	JOND920102 (10)	SNEP660101 (9)
2	FUKS010106 (7)	DESM900101 (7)
3	MCMT640101 (6)	KYTJ820101 (6)
4	MEEJ810101 (6)	WOLS870102 (6)
5	BEGF750103 (5)	JOND920102 (6)
6	KYTJ820101 (4)	FUKS010106 (5)
7	ROBB790101 (4)	BEGF750103 (3)
8	KIDA850101 (3)	CORJ870108 (3)
9	ROBB760108 (3)	LEVM780106 (2)
10	MIYS990101 (3)	AURR980120 (2)

The description of each AAindex ID can be found at http://www.genome.jp/dbget-bin/www_bfind aaindex.

**Figure 4:**
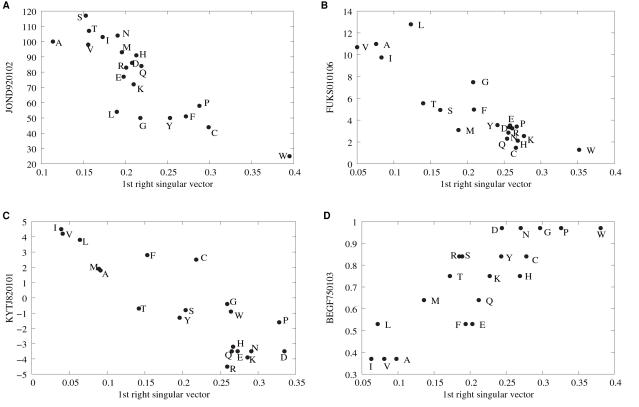
Examples of correlations between the first r-SV and amino acid indices. The abscissa of each panel indicates the value of elements in the first right singular vector of a PSSM where the signs are determined by making the value for cysteine positive. The ordinates are (A) the relative mutability [25], (B) the interior composition of amino acids in intracellular proteins of mesophiles[26], (C) the hydropathy scale[28], and (D) the conformational parameter of beta-turn[27]. The labels on the ordinates indicate the identifiers of the AAindex database (Table 1).

In the PDB representative set, the most frequently correlated index was the relative mutability[25] (AAindex: JOND920102) which is also the fifth most frequent index for the Pfam set. The relative mutabilities[5] represent the tendency of amino acid residues to be mutated during molecular evolution, and are not highly correlated with any other indices [8]. It is thus expected that some intrinsic characteristics of protein evolution is embedded in their values. The relative mutability is the most dominant component in the ordinary (position-independent) AASMs targeted at closely related proteins[9]. As in the case of AASMs, the first r-SVs are negatively correlated with the relative mutability (Recall that all the elements of the first r-SVs are of the same sign in most cases so that we can make them all positive without losing generality). An example is shown in Fig. 4 A. Thus, noting that the first singular components (i.e., partial matrix *M*_1_ in Fig. 3) are mostly negative, we can see that substitutions of those residues with low mutabilities are more severely penalized.

The interior composition of amino acids in intracellular proteins of mesophiles[26] (FUKS010106) is another frequently correlated index, ranked second and sixth in the PDB and Pfam sets, respectively. As we can see in the example shown in Fig. 4 B, those residues that are less abundant in protein interior are more severely penalized. This seems to contradict our intuition that residues in the protein interior are more conservative than those on the protein surface. However, many functionally important residues exist on the surface (ligand binding sites and catalytic sites, etc.). Thus, these r-SVs should be regarded as representing potentially functionally important residues. Note, however, although these residues share some properties common to conserved residues, most of them are not actually important (otherwise they should not be penalized).

Other frequently correlated indices shared by both PDB and Pfam sets are the conformational parameter of β-turn[27] (BEGF750103) and the hydropathy index of Kyte and Doolittle[28] (KYTJ820101). The most frequently correlated index for the Pfam set was “principal component I” of Sneath (SNEP660101) [29]. The name of this index is rather cryptic, but it is weakly negatively correlated with turn or coil propensities (data not shown). These indices can be readily related to interior-surface propensities: β-turns, coils, and hydrophilic residues tend to be on the surface of a protein, and so on. The general trend is that substitutions of those residues that tend to be on the surface are more severely penalized (Fig. 4C, D). Again, this may be due to the fact that many (potentially) functionally important residues are on the protein surface.

It is noted that no single index is overwhelmingly dominant in the first r-SVs so that different PSSMs are characterized by different properties. This is a reasonable result since each PSSM is specific to a particular protein family which is under the influence of specific evolutionary pressures and biological constraints. Nevertheless, relative mutability, hydrophobicity, and turn/coil propensity are the relatively more dominant characteristics of the first r-SVs.

**Figure 5:**
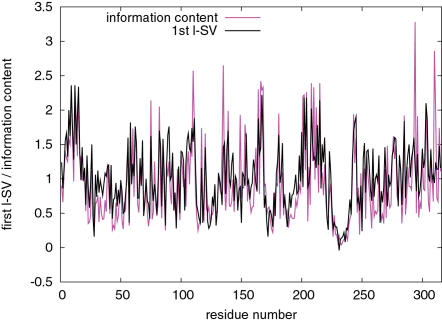
Example of the first left singular vector and information content. Shown are the first l-SV and information content of the PDB entry 1e6uA[60]. The values of the l-SV elements are scaled by 20 times to match the information content. The correlation coefficient is 0.76.

If the first r-SV of a PSSM represents a property of amino acid residues that is well-conserved, then the first l-SV is expected to represent the pattern or extent of conservation of that property along the amino acid sequence. One such measure is the information content (also referred to as Kullback-Leibler divergence or relative entropy [30]) which is a kind of distance of the distribution of amino acid residues at a given site of the sequence from the background distribution. The information content *D_i_* of site *i* is defined as
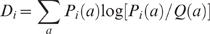
(4)where *P_i_*(*a*) is the frequency of amino acid type *a* at the site *i* and *Q*(*a*) is the background frequency of amino acid type *a*. In general, information content tends to be larger at more conserved sites. This information is available in the PSSMs created with PSI-BLAST. A significant correlation was found between the first l-SVs and information content of PSSMs of the PDB set with correlation coefficient of 0.543 on average with standard deviation of 0.218 (*P*<10^−17^, assuming the average sequence length of 217 residues). The median of the correlation coefficient was 0.601 indicating that the correlation is even higher for many of the PSSMs. When calculating the correlation coefficient, we converted the signs of the elements of the l-SV so that most elements become positive. Thus, a positive correlation implies that a site with a large value of the first l-SV element usually has high information content, indicating that substitutions at those sites with more information content are more severely penalized. An example of such correlation is shown in Fig. 5. l-SVs other than the first one did not show high correlations with information content (data not shown). For those PSSMs whose first r-SVs are highly correlated with JOND920102 (110 entries), FUKS010106 (74), BEGF750103 (56), KYTJ820101 (49), and SNEP660101 (24) (Table 2), the average correlation coefficients were 0.646, 0.703, 0.654, 0.536, and 0.593, respectively. Thus, the high correlation between the first l-SV and information content is not limited to specific PSSMs whose first r-SVs are correlated to some particular indices.

### Characteristics of second singular components

In the same manner as the first r-SVs, we searched for indices that are highly correlated with the second r-SVs of the PSSMs (Table 3). In this case, relative partition energies derived by the Bethe approximation of Miyazawa and Jernigan [31] (AAindex: MIYS990101) is the most correlated index: 33% of the PDB set and 54% of the Pfam set. This index is a kind of hydrophobicity scale. Furthermore, other frequently correlated indices, such as interactivity scales of Bastolla et al. [32] (BASU050101, BASU050103), polarity [33] (GRAR740102), optimal matching hydrophobicity [34] (SWER830101), and all other indices in Table 3, are all related to hydrophobicity scales. The ten most frequently correlated indices alone match 85% and 94% of the second r-SVs of the PSSMs in the PDB and Pfam sets, respectively. Therefore, while the first r-SVs are of diverse characteristics, the second r-SVs are almost exclusively determined by hydrophobic properties. It is interesting to note that the hydropathy index of Kyte and Doolittle [28] which was found to be correlated to some first r-SVs (Table 2) was not found to be the the index most correlated with the second r-SVs in most cases. Although the hydropathy index is highly correlated with the partition energy of Miyazawa and Jernigan (correlation coefficient of −0.84), there seems to be a meaningful difference between them.

**Table 3:** Amino acid indices most correlated to the second right singular vectors [frequency (%) in the parentheses].

rank	PDB	Pfam
1	MIYS990101 (33)	MIYS990101 (54)
2	BASU050101 (12)	GRAR740102 (12)
3	BASU050103 (11)	BASU050103 (10)
4	GRAR740102 (8)	MIYS990102 (7)
5	SWER830101 (7)	BASU050101 (6)
6	MIYS990102 (6)	SWER830101 (1)
7	ZHOH040103 (2)	ZHOH040103 (1)
8	CORJ870102 (2)	MIYS990105 (1)
9	KYTJ820101 (2)	FAUJ830101 (1)
10	FAUJ830101 (2)	CORJ870102 (1)

**Figure 6:**
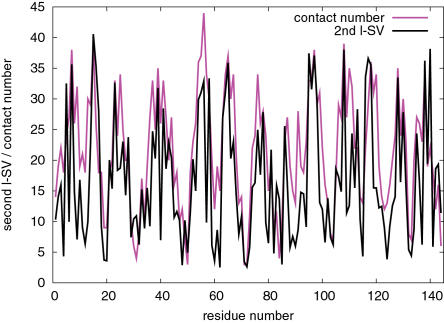
Example of the second left singular vector and contact numbers. Shown are the second l-SV and contact numbers of the PDB entry 1l2hA[61]. The values of the l-SV elements are shifted and scaled to match the contact numbers. The correlation coefficient is 0.71.

The correlation between the second r-SVs and hydrophobicity scales is striking. Therefore, it is expected that the second left singular vectors (l-SVs) are correlated with some structural property that is dual to the hydrophobicity. One such structural property is the contact number [35], [36], [37], which is the number of residues in contact with a given residue in a native protein structure. We calculated contact numbers of the PDB set (based on the definition by Kinjo et al. [37]) and their correlations with the second l-SVs. The average correlation coefficient was 0.511 (standard deviation 0.113) which is highly significant (*P*<10^−15^) for the average protein length of 217 residues in the PDB set. Fig. 6 shows an example of the highly correlated second l-SV and contact numbers.

Recall that the elements of the first r-SVs were of the same sign in most cases (Fig. 3). Thus, by the orthogonality of singular vectors, the elements of the second r-SVs should necessarily contain values of both signs in most cases. The same argument also applies to l-SVs. Therefore, the contribution from the second component of a PSSM, namely 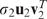, contains both positive and negative elements corresponding to favorable and unfavorable substitutions, respectively. Now let **w** represent the relative partition energy of Miyazawa and Jernigan [31] (MIYS990101), and **n** represent the contact number vector of a protein standardized by subtracting the average value from each element. We calculated the correlation coefficient between the two matrices 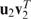 and **wn** *^T^* for those 361 proteins whose second r-SVs are most correlated with **w**. We obtained the average correlation of −0.45 which is highly significant (*P*<10^−220^) taking into account the average number of elements (217×20). Since hydrophilic and hydrophobic residues have positive and negative partition energies, respectively, the negative correlation means that hydrophobic residues with high contact numbers (buried) and hydrophilic residues with low contact numbers (exposed) are more favored compared to hydrophobic residues with low contact numbers and hydrophilic residues with high contact numbers. Thus, within the framework developed here, we can consider the second singular component represents the structural stability of the protein.

### Characteristics of third and other singular components

The indices that are most frequently correlated with the third r-SVs of the PSSMs are listed in Table 4. In general, the third r-SVs are correlated with those indices related to the volume or bulkiness of amino acid residues such as CHAM830106, SNEP660103, LEVM760102, LEVM760105 and OOBM770105 (see Table 1 for descriptions). Another kind of index common to the PDB and Pfam sets is the α-helix propensity derived from designed sequences[38] (KOEP990101) which is actually correlated with coil propensity (data not shown). This index was also found to be frequently correlated with the fourth r-SVs. A structural quantity that may be associated with bulkiness of amino acid residues is the volume of the “territory” of residues as defined by the Voronoi tessellation[39], [40]. When we compared the Voronoi volumes calculated from protein structures with the third l-SV, we observed a significant but weak correlation of 0.345 (*P*<0.0003). (The Voronoi volume of a residue was calculated by summing the Voronoi volumes of the atoms that belong to the residue; only half of the residues with smaller volumes are used for comparison as surface residues with [sometimes infinitely] large volumes are not meaningful.) If we limit the comparison to those proteins whose third r-SVs are most correlated with CHAM830106 (214 entries), SNEP660103 (201) or LEVM760102 (137), the correlations were 0.366, 0.251, or 0.479, respectively. Therefore, the correlation of the third l-SV to the Voronoi volume is significant, but not as consistent as those of the first and second l-SVs to information content and contact numbers, respectively.

**Table 4:** Amino acid indices most correlated to the third right singular vectors [frequency (%) in the parentheses].

rank	PDB	Pfam
1	CHAM830106 (20)	CHAM830106 (15)
2	SNEP660103 (18)	SNEP660103 (15)
3	LEVM760102 (12)	LEVM760102 (12)
4	KOEP990101 (8)	LEVM760105 (8)
5	OOBM770105 (6)	WOLS870102 (5)
6	LEVM760105 (6)	FASG760101 (5)
7	MITS020101 (6)	KOEP990101 (4)
8	HUTJ700103 (2)	CHAM830104 (4)
9	RADA880103 (2)	HUTJ700103 (3)
10	CHAM830105 (2)	OOBM770105 (3)

The propensity of the fourth and fifth r-SVs are not so clearly characterized as the first three r-SVs, but helix (KOEP990101) and helix cap propensities [41] as well as some bulkiness parameters are relatively highly correlated with the fourth r-SVs, while the net charge (KLEP840101) [42] and α-NH chemical shifts (BUNA790101) [43] were the indices most correlated with the fifth r-SVs of more than 30% of the PSSMs in both the PDB and Pfam sets.

### Example: Conserved sites in the globin family

**Figure 7:**
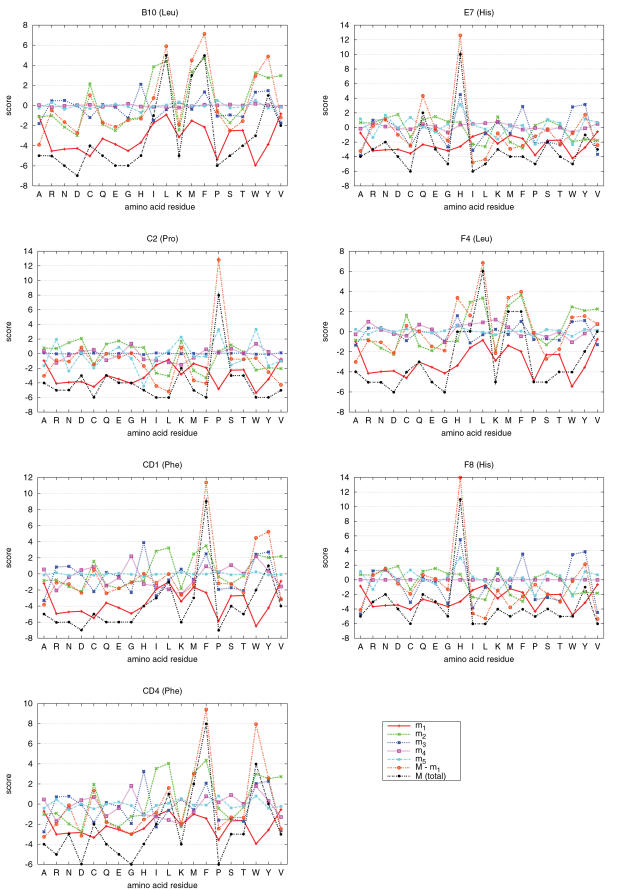
Decomposed PSSM scores of conserved sites in the globin family. The labels B10, C2, CD1, CD4, E7, F4, and F8 on the top of panels are site identifiers of the globin family defined by Bashford et al.[45] (in the parentheses is the most conserved residue at each site). Rows of the partial matrices 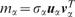 (α = 1, …, 5) as well as *M*−*m*_1_ are plotted for the selected sites. The PSSM is based on the PDB entry 3sdhA[44].

To illustrate the points made above, we now examine the PSI-BLAST PSSM of a globin (PDB 3sdhA[44], hemoglobin I from *Scapharca inaequivalvis*). The globin family is one of the most extensively studied protein families[45], [46]. Ota et al.[47] examined in detail seven highly conserved residues in globins identified by Bashford et al.[45] (namely, the sites B10, C2, CD1, CD4, E7, F4, and F8, according to the numbering scheme of Bashford et al.[45]), and succeeded in separating structurally important sites from functionally important sites. Fig. 7 shows the contributions of various components to the seven highly conserved sites studied in Ota et al. [47]. The most correlated amino acid indices for the first 5 r-SVs are BEGF750103, MIYS990101, FAUJ880106, KOEP990101, and AURR980119 (see Table 1 for their descriptions).

The contributions to those conserved residues that were identified as functionally important by Ota et al. [47] (namely, E7 and F8) are mainly from the third and fifth components which are correlated to bulkiness and helix capping propensity, respectively. Other conserved residues were identified as structurally important, and their scores consist mainly of the second singular component which is related to the hydrophobicity, except for the proline residue at the C2 site to which the helix capping propensity is the main contributor. These observations are consistent with the analysis of Ota et al. [47] which was based on three-dimensional profiles[48], [49].

The contributions of the first singular component to these sites are all negative for all residues (Fig. 7) which is consistent with the general argument provided above. We now consider the meaning of the negative contribution of the first singular component. For simplicity, we first consider the site F8 where the histidine residue is perfectly conserved. At this site, only the score of histidine should be positive and all others be negative. Positive contributions to the score of histidine is made from the third, fifth and other singular components so that the total contributions from second to twentieth components are as large as 14. Without the contribution from the first singular component, the scores of some other residues such as asparagine and tyrosine are also positive although not as large as that of histidine. Thus, we can see that the large positive score of a conserved residue (histidine) is made by coherent contributions from multiple singular components whereas the scores of residues that are not conserved may be positive but small due to incoherent contributions. Nevertheless, positive scores of non-conserved residues degrades the specificity of a PSSM. Thus, they should be somehow made negative. Similar arguments apply to other conserved sites except that different residues may be conserved at different sites for different reasons. The score of potentially but falsely functionally important residues at all conserved sites can be made negative at once by simply subtracting the scores according to the common properties of amino acid residues at these sites, and this is the role of the first singular component. In the present example, the common property happened to be related to the β-turn propensity.

Note that the shapes of the corresponding components (e.g., *m*_1_ for different sites) are similar among different sites. This is because they are all scalar multiple of the same r-SVs. What distinguishes different sites is the relative contributions due to the l-SVs.

## Discussion

Kinjo and Nishikawa[9] analyzed a set of amino acid substitution matrices constructed from multiple alignments of protein families of varying percent sequence identities (%ID). It was found that, at high %IDs (>35%), the first and second most dominant components were correlated with relative mutability and hydrophobicity, respectively, while at low %IDs (<30%), the order was opposite (hydrophobicity first, and then the relative mutability). It was suggested that the dominance of the relative mutability over hydrophobicity patterns is the prerequisite for reliable detection of homologs. In the case of PSSMs, the characteristics of the first singular component may vary depending on the protein (family). Nevertheless, the first singular components seem to represent some functional constraints which disfavor any substitutions, and the second (and third) singular components are predominantly determined by such structural requirements as hydrophobicity (and packing). Although both functional and structural constraints are important for distant homolog detection, the dominance of the former over the latter may be more influential for the high specificity of sequence alignment methods based on PSSMs. Noting again that the Pfam PSSMs have larger first singular values (Fig. 2) and their first components contain more negative elements (Fig. 3) compared to PSI-BLAST-generated PSSMs of the PDB set, this view of the first singular component is consistent with a general observation that Pfam PSSMs exhibit, on average, higher specificity than those generated by PSI-BLAST.

As pointed out by Tomii and Kanehisa [8], side-chain volume and hydrophobicity are the main ingredients of AASMs. In addition to these two properties, Pokarowski et al.[10] also noted the importance of the coil propensity. Wrabl and Grishin[50] also found similar preferences in the study of properties extracted from multiple sequence alignments. These properties are also found to be the main ingredients of PSSMs in the present study. Some of the first r-SVs showed significant correlation to indices related to coil propensity such as BEGF750103 and SNEP660101 (Table 2); hydrophobicity is predominant in the second r-SVs; and side-chain volumes often show high correlation with the third r-SVs.

Bastolla et al.[32] have studied the correlation between the “interactivity” scale of amino acid residues and the principal eigenvectors of the native contact maps[51]. Their interactivity scale is a kind of hydrophobicity scale, obtained by eigenvalue decomposition of a contact potential and subsequent optimizations. The principal eigenvector of a contact map is known to contain almost sufficient information for recovering the native structure itself[51], and is highly correlated with contact number vector [52]. Bastolla et al.[32] showed that the interactivity scales aligned along the amino acid sequence of a protein, then averaged over homologs, were significantly correlated with the principal eigenvector with the average correlation coefficient of 0.47. Note that the interactivity scales of Bastolla et al. are found among those indices that are most correlated with the second r-SVs in Table 3 (BASU050101 and BASU050103), and that the second l-SVs are correlated with contact number vectors. Thus, the present result is not only consistent with that of Bastolla et al. [32], but also demonstrates that some structural information is already embedded in a PSSM, which also explains why contact numbers can be predicted at high accuracy by using PSSMs[37], [53], [54], [55], [56]. The present finding may be useful for deriving optimized contact potentials[57].

To summarize, we analyzed PSSMs by decomposing them into singular components (Table 5). The characteristics of the first right singular vectors was found to vary depending on protein families, but the corresponding left singular vectors showed high correlation with information content. The contributions of the first singular components to the original PSSMs are usually negative so that the substitutions of potentially but falsely functionally important residues at conserved sites are more severely penalized. The second right singular vectors were almost always related to hydrophobicity of amino acid residues, and the left singular vectors are significantly correlated with contact number vectors, thus demonstrating that the structural information is directly embedded in the PSSMs. Other structural information seem to be also included in the PSSMs, although not as significantly as hydrophobicity and contact numbers. Therefore, sequence alignment using PSSMs may be regarded as threading [58], [48], [59] supplemented with some functional information. Based on the present analysis, it may be possible to define *a priori* measure of the quality of PSSMs which may lead to a rational strategy for constructing more effective PSSMs by mixing various functionally/structurally relevant contributions with appropriate singular values.

**Table 5:** Summary of characteristics of first three singular vectors of PSSMs.

component	left SV	right SV	Remark
1	information content	relative mutability, etc.	negative contribution
2	contact number	hydrophobicity	structural stability
3	Voronoi volume	bulkiness	weak correlation
